# Lefamulin. Comment on: “Novel Antibiotics for Multidrug-Resistant Gram-Positive Microorganisms. *Microorganisms*, 2019, *7*, 270”

**DOI:** 10.3390/microorganisms7100386

**Published:** 2019-09-24

**Authors:** Despoina Koulenti, Elena Xu, Isaac Yin Sum Mok, Andrew Song, Drosos E. Karageorgopoulos, Apostolos Armaganidis, Jeffrey Lipman, Sotirios Tsiodras

**Affiliations:** 1 UQ Centre for Clinical Research, Faculty of Medicine, The University of Queensland, Brisbane, QLD 4072, Australia; elena.xu@uq.net.au (E.X.); isaac.mok1@uq.net.au (I.Y.S.M.); a.song@uq.net.au (A.S.); j.lipman@uq.edu.au (J.L.); 2 2nd Critical Care Department, Attikon University Hospital, 12462 Athens, Greece; aarmag@med.uoa.gr; 3 4th Department of Internal Medicine, Attikon University Hospital, 12462 Athens, Greece; drkarag@gmail.com (D.E.K.); sotirios.tsiodras@gmail.com (S.T.); 4 Department of Intensive Care Medicine, Royal Brisbane and Women’s Hospital, Brisbane 4029, Australia; 5 Anesthesiology and Critical Care, Centre Hospitalier Universitaire De Nîmes (CHU), University of Montpellier, 30029 Nîmes, France

**Keywords:** multidrug-resistance, Gram-positive pathogens, novel antibiotics, pleuromutilins, lefamulin

## Abstract

On 18 August 2019, an article was published in *Microorganisms* presenting novel, approved anti-Gram-positive antibiotics. On 19 August 2019, the U.S. Food and Drug Administration announced the approval of lefamulin, a representative of a new class of antibiotics, the pleuromutilins, for the treatment of adult community-acquired bacterial pneumonia. We present a brief description of lefamulin.

## Introduction

On 18 August 2019, an article was published in *Microorganisms* presenting novel, approved anti-Gram-positive antibiotics [1]. On 19 August 2019, the U.S. Food and Drug Administration (FDA) announced the approval of lefamulin for the treatment of adult community-acquired bacterial pneumonia (CABP) [2]. 

## Lefamulin

Lefamulin (formerly known as BC-3781), developed by Nabriva Therapeutics, is a semi-synthetic pleuromutilin mainly studied for CABP and acute bacterial skin and skin-structure infections (ABSSSIs) [3]. Lefamulin has a novel mechanism of action of selective binding to the peptidyl transferase center (PTC) of the 50S ribosomal subunit to prevent bacterial protein synthesis [3,4]. The spectrum of activity of lefamulin covers Gram-positive pathogens including methicillin-resistant *Staphylococcus aureus* (MRSA), as well as *Moraxella catarrhalis, Haemophilus influenzae*, and atypical pathogens such as *Mycoplasma pneumoniae, Chlamydophila pneumoniae*, and *Legionella pneumophila* [5,6,7]. An in vitro study that assessed the antimicrobial activity of lefamulin against common pathogens causing CABP demonstrated lefamulin to have the lowest minimum inhibitory concentration (MIC) against penicillin non-susceptible and macrolide-resistant *Streptococcus pneumoniae* among its comparators. Moreover, lefamulin was found to be not susceptible to cross-resistance from other antibiotic classes [5]. 

In terms of its pharmacokinetic properties, lefamulin exhibits high nonlinear plasma protein binding with low unbound concentrations, higher concentrations in lung epithelial lining fluid (ELF) than in plasma, and a half-life of approximately 10 h [8]. It can be administered either as 150 mg every 12 h by intravenous infusion (IV) over 60 min for 5 to 7 days, or as 600 mg orally every 12 h for 5 days [2]. Its oral bioavailability when administered on an empty stomach is about 25%. Lefamulin is distributed rapidly into the interstitial space of skeletal muscle, subcutaneous adipose tissue, and lung ELF after a single dose of a 150 mg IV infusion [8].

Two phase III studies (LEAP1 and LEAP2 trials) have tested the safety and efficacy of lefamulin in adult patients with CABP [9,10]. LEAP1 (NCT02559310) was a multicenter, double-blind randomized trial conducted in adult patients with CABP and a Pneumonia Outcomes Research Team (PORT) risk class ≥III [9]. LEAP1 study patients were randomized 1:1 to receive either lefamulin 150 mg IV every 12 h or moxifloxacin 400 mg IV every 24 h combined with adjunctive linezolid for any patient with suspected MRSA, with subsequent switching to oral lefamulin and oral moxifloxacin [9,11,12]. The results of the study showed that lefamulin was safe and well-tolerated [11,12]. It was found to be noninferior to moxifloxacin in the early clinical response (ECR), namely, 96 ± 24 h after the first dose of the study drug in the intent-to-treat (ITT) population that was FDA’s primary endpoint (87.3% vs. 90.2%, respectively) [11,12]. In addition, it was noninferior to moxifloxacin in the European Medicines Agency (EMA)’s coprimary endpoints, i.e. the investigator assessment of clinical response (IACR) 5–10 days after last dose of the study drug in the modified ITT (mITT) population (81.7% vs. 84.2%, respectively) and in the clinically evaluable (CE) population (86.9% vs. 89.4%, respectively) [11,12]. LEAP2 (NCT02813694) was another multicenter, randomized, double-blind study that compared the safety and efficacy of oral lefamulin (dose of 600 mg every 12 h for 5 days) with that of moxifloxacin (dose of 400 mg every 24 h for 7 days) in patients with CABP and PORT risk class between II and IV [10,13]. This study aimed to complement the results of the LEAP1 study [10,13]. It was reported that lefamulin was noninferior to moxifloxacin regardless of the PORT risk class for both FDA’s and EMA’s endpoints, i.e. for ECR in the ITT population which was the FDA’s primary endpoint (for PORT II, III, IV: 91.8%, 91.0%, and 85.0% for lefamulin; 93.1%, 90.2%, and 85.7% for moxifloxacin, respectively), and for the EMA’s coprimary endpoints of IACR in mITT population (87.5% vs. 89.1%, respectively) and in CE-test-of-cure (TOC) population (89.7% vs. 93.1%, respectively) [13]. Serious adverse events did not differ between the two groups, while the rate of discontinuation was low and similar in both groups [13]. 

A multicenter, double-blind, randomized, parallel-group, phase II study (NCT01119105) compared lefamulin (100 mg) and lefamulin (150 mg) with a vancomycin (1 g) IV infusion every 12 h for 5 to 14 days for ABSSSIs caused by Gram-positive pathogens [14]. The presence of MRSA along with other clinical characteristics were comparable between the groups [14]. It was found that lefamulin had a clinical success rate comparable to vancomycin, i.e., in the CE population the TOC rates for lefamulin 100 mg, 150 mg, and vancomycin were 90%, 88.9%, and 90.2%, respectively [15]. Lefamulin was well-tolerated and both lefamulin doses (100 mg and 150 mg) had fewer adverse events than vancomycin (34.3%, 39.4%, and 53%, respectively) [15]. 

The commonest adverse drug reactions from the IV formulation include injection site reactions (7%), hepatic enzyme elevation (3%), nausea (3%), hypokalemia (3%), insomnia (3%), and headache (2%) [2]. The oral formulation commonly causes diarrhea (12%), nausea (5%), vomiting (3%), and hepatic enzyme elevation (2%) [2]. Intravenous lefamulin requires dose adjustment in severe hepatic impairment (Child–Pugh Class C), specifically by extending the dosing interval [2]. Oral lefamulin has not been studied and is therefore not recommended in those patients with moderate or severe liver impairment (Child–Pugh Classes B and C) [2]. Dosage adjustment for renal impairment, including patients on hemodialysis, is not required as lefamulin predominantly utilizes the hepatobiliary pathway for excretion (15.5% and 5.3% unchanged in urine after 150 mg IV and 600 mg oral dose, respectively) [2].

The chemical structure and further information on lefamulin can be found in the electronic Supplementary Materials.

## Supplementary Materials

The following are available online at https://www.mdpi.com/2076-2607/7/10/386/s1.
