# Supplementary material for: Lefamulin. Comment on: “Novel Antibiotics for Multidrug-Resistant Gram-Positive Microorganisms. Microorganisms, 2019, 7, 270”
Source: Microorganisms. 2019 Sep 24;7(10):386. doi: 10.3390/microorganisms7100386 (PMC6843636; doi:10.3390/microorganisms7100386)
Supplement: Supplementary file 1 [file microorganisms-07-00386-s001.pdf]

Supplement

Reference: National Center for Biotechnology Information. PubChem Database. Lefamulin, CID=25185057, https://pubchem.ncbi.nlm.nih.gov/compound/Lefamulin [accessed 15/09/2019]

Lefamulin

Chemical name: Lefamulin
PubChem CID: 25185057
Molecular Formula: $C_{28}H_{45}NO_5S$
Molecular Weight: 507.7 g/mol
2D Structure:

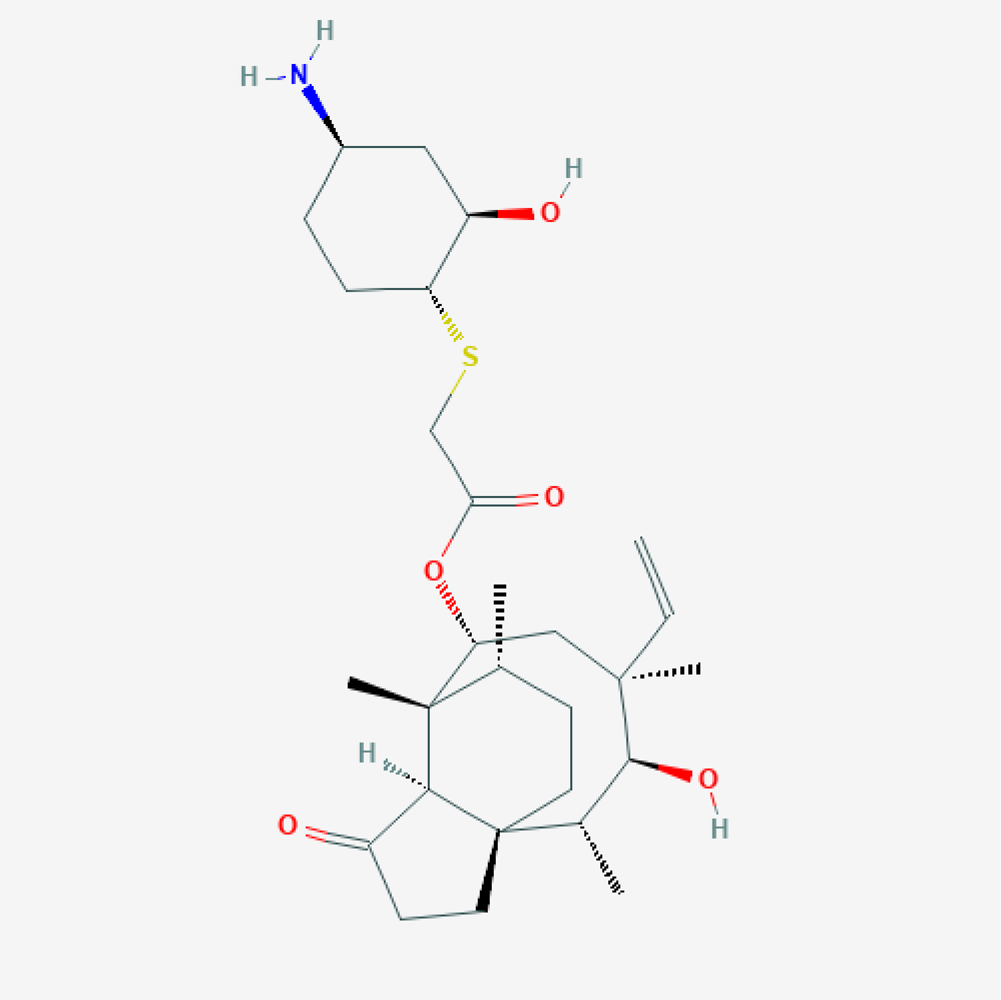

IUPAC Name:
[(1*S*,2*R*,3*S*,4*S*,6*R*,7*R*,8*R*,14*R*)-4-ethenyl-3-hydroxy-2,4,7,14-tetramethyl-9-oxo-6-tricyclo[5.4.3.0^{1,8}]tetradecanyl] 2-[(1*R*,2*R*,4*R*)-4-amino-2-hydroxycyclohexyl]sulfanylacetate
